# UVB Inhibits Proliferation, Cell Cycle and Induces Apoptosis via p53, E2F1 and Microtubules System in Cervical Cancer Cell Lines

**DOI:** 10.3390/ijms22105197

**Published:** 2021-05-14

**Authors:** Angelica Judith Granados-López, Eduardo Manzanares-Acuña, Yamilé López-Hernández, Julio Enrique Castañeda-Delgado, Ixamail Fraire-Soto, Claudia Araceli Reyes-Estrada, Rosalinda Gutiérrez-Hernández, Jesús Adrián López

**Affiliations:** 1 Laboratorio de microRNAs y Cáncer, Unidad Académica de Ciencias Biológicas, Campus II, Universidad Autónoma de Zacatecas, Av. Preparatoria S/N, Zacatecas 98068, Mexico; agranadosjudith@gmail.com (A.J.G.-L.); ixamail13@gmail.com (I.F.-S.); 2 Unidad Académica de Estudios Nucleares, Campus II, Universidad Autónoma de Zacatecas, Av. Preparatoria S/N, Zacatecas 98068, Mexico; emanz_44@yahoo.com; 3 CONACYT, Laboratorio de Metabolómica y Proteómica, Unidad Académica de Ciencias Biológicas, Campus II, Universidad Autónoma de Zacatecas, Av. Preparatoria No.301, Zacatecas 98068, Mexico; ylopezher@conacyt.mx; 4 Catedrático-CONACYT, Unidad de Investigación Biomédica de Zacatecas, Instituto Mexicano del Seguro Social, Zacatecas 98000, Mexico; jecastanedade@conacyt.mx; 5 Laboratorio de Inmunohistoquímica y EOx, MCS de la Unidad Académica de Medicina Humana, Campus Siglo XXI, Universidad Autónoma de Zacatecas, Kilómetro 6, Ejido la Escondida, Zacatecas 98160, Mexico; c_reyes13@uaz.edu.mx; 6 Laboratorio de Etnofarmacología Nutrición, Unidad Académica de Enfermería, Campus Siglo XXI, Universidad Autónoma de Zacatecas, Kilómetro 6, Ejido la Escondida, Zacatecas 98160, Mexico; rosalinda@uaz.edu.mx

**Keywords:** UVB irradiation, microtubule system, immortal and tumorigenic cells, p53 expression, E2F1 expression

## Abstract

Ultraviolet (UV) exposure has been linked to skin damage and carcinogenesis, but recently UVB has been proposed as a therapeutic approach for cancer. Herein, we investigated the cellular and molecular effects of UVB in immortal and tumorigenic HPV positive and negative cells. Cells were irradiated with 220.5 to 1102.5 J/m^2^ of UVB and cell proliferation was evaluated by crystal violet, while cell cycle arrest and apoptosis analysis were performed through flow cytometry. UVB effect on cells was recorded at 661.5 J/m^2^ and it was exacerbated at 1102.5 J/m^2^. All cell lines were affected by proliferation inhibition, cell cycle ablation and apoptosis induction, with different degrees depending on tumorigenesis level or HPV type. Analysis of the well-known UV-responsive p53, E2F1 and microtubules system proteins was performed in SiHa cells in response to UVB through Western-blotting assays. E2F1 and the Microtubule-associated protein 2 (MAP2) expression decrease correlated with cellular processes alteration while p53 and Microtubule-associated Protein 1S (MAP1S) expression switch was observed since 882 J/m^2^, suggesting they were required under more severe cellular damage. However, expression transition of α-Tubulin3C and β-Tubulin was abruptly noticed until 1102.5 J/m^2^ and particularly, γ-Tubulin protein expression remained without alteration. This study provides insights into the effect of UVB in cervical cancer cell lines.

## 1. Introduction

Cervical cancer (CC) is the fourth most common cancer in women worldwide, with an estimated global incidence of 604,127 new cases and over 341,831 deaths per year [1]. High-risk human papillomavirus (HPV) infection has been associated with the development of cervical, penile and anal cancers. Ionizing radiation through high energy X- or γ-rays is usually used during CC treatment. Ionizing radiation has a strong penetrance and possesses an intrinsic capacity to elicit new malignant cells in the vicinity of irradiated cancerous tissue. UV radiation has been compared with γ irradiation in its capability to elicit cell death. Interestingly, it has been shown that UV radiation in shorter time than γ radiation induces apoptosis in a p53 dependent manner [2,3,4]. UV light is currently being used to treat cutaneous T-cell lymphoma [5]. Therefore, UV could be used as a therapeutic agent as effective dose and wavelength dependent. Apoptosis induction via UV irradiation (UV-IR) has been well documented in a variety of cell types in a p53 dependent and independent manner, and thus p53 may mediate some of the UV-induced programmed cell death [6,7].

p53 and the retinoblastoma protein (pRb) tumor suppressor proteins are down-regulated via HPV E6 and E7 oncoproteins, respectively [8]. HPV E6 protein binds to cellular Human Papilloma Virus E6-Associated Protein (E6AP) protein, enabling proteasomal p53 degradation, while E7 protein binds to the hypophosphorylated form of Rb, disrupting the complex between Rb and the transcription factor E2F-1, resulting in E2F-1 release and allowing the transcription of genes required for the cell to access the cell cycle S phase [9]. Subsequently allowing DNA mutation and cellular transformation lead to the unavoidable cancerous cells’ characteristics of unlimited proliferation, apoptosis inhibition, migration and invasion capacity, which require high cellular activity and plasticity. Cancer cell activity may be improved by cell protein trafficking and cellular structural changes, where cytoskeleton structures participate through microtubules and their associated proteins.

Microtubules are dynamic structures, participating in mitosis, cell cycle, movement and intracellular trafficking; thus, their function and associated proteins may be closely related with cancer cells dynamics and therefore are an object of study in the identification of cancer therapy targets. γ-tubulin is a protein that plays a role in microtubule nucleation, assembly and polarity establishment [10]. In cytosol, γ-tubulin nucleates α- and β-tubulin into a growing microtubule. Nuclear γ-tubulin regulates S-phase progression by moderating the activity of E2F1 [11]. Thus, tubulins and microtubule-associated proteins (MAPs) may play a role in cellular stress responses [12]. MAP2 is a protein associated with microtubule stabilization and it binds to filamentous actin, and participates in the recruitment of signal proteins and the regulation of microtubule-mediated transport [13]. MAP2 over-expression has been shown in malignant diseases such as cutaneous melanoma [14], pulmonary carcinoid tumor and small cell carcinoma [15] and it has been correlated with cell motility and invasion in epithelial and oral cancer cells [16]. MAP1S, a microtubule-associated protein, has been implicated in microtubule dynamics, mitotic abnormalities and mitotic cell death. On the other side, MAP1S positively regulated the expression of Bcl-2, B-cell lymphoma-extra large (Bcl-xL) and P27 protein levels, the distribution of mitochondria and several responses in nutritive stress [17]. Essential roles of microtubules and microtubule-associated proteins include chromosome segregation and nucleus positioning during mitosis. Thus, defects in these functions may lead to proliferation inhibition and cell death in tumor cells [18]. In this work, we evaluated the proliferation, cell cycle, apoptosis and protein expression of the well-known marker of UV-IR response, p53; cell cycle transcriptional regulator, E2F1; and proteins of the microtubules system (MAP1S, MAP2, α, β, and γ-Tubulin) in response to UVB. In this work, we show that tumorigenic status and lack of HPV may provide UVB susceptibility to cells than tumorigenic HPV positive and/or immortal cells. Protein expression in the SiHa cell line was UVB dose-dependent. E2F1 and MAP2 proteins were regulated since lower levels of UVB dose than p53 and MAP1S, while tubulin regulation was evident only at higher doses of UVB. This work sheds light on potential protein biomarkers for cervical cancer therapy using UVB radiation.

## 2. Results

### 2.1. UVB Inhibits Proliferation of Immortal, Tumorigenic and HPV-Positive and Negative Cells in A Dose-Dependent Manner

**Figure 1:**
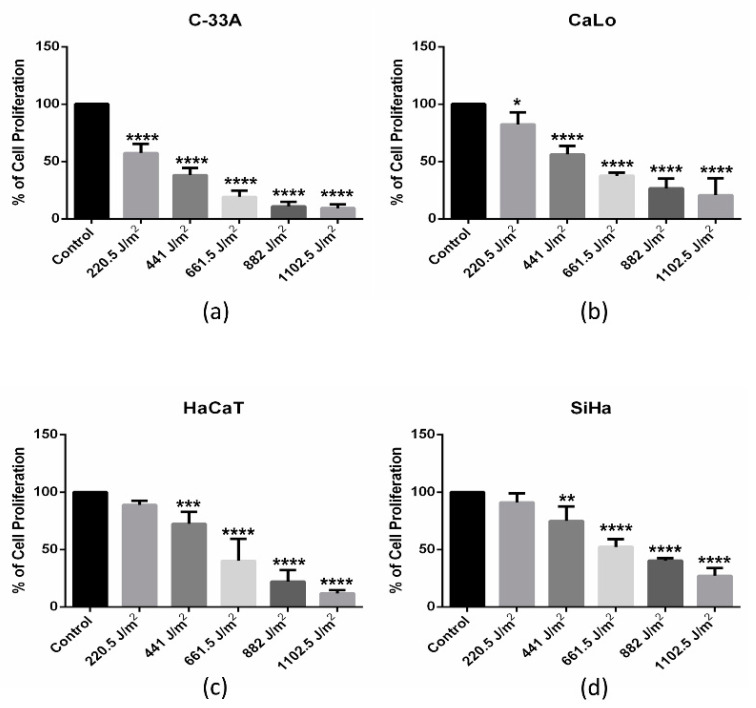
UVB inhibits proliferation of cervical cancer cell lines in a dose-dependent manner. (**a**) C-33A, (**b**) CaLo, (**c**) HaCaT and (**d**) SiHa cells were treated with increasing doses of UVB. After 72 h of incubation, cellular proliferation was analyzed. Bars represent mean and standard deviation of three independent experiments (*p* < 0.05) * = 0.0415, ** = 0.0047, *** = 0.0002, **** = <0.0001. Control cells were not UVB irradiated.

Cell tumorigenesis status as well as HPV infection may be determinant in the susceptibility or resistance to UVB irradiation. In this work, we evaluated the proliferation effect on immortal HPV negative (HPV-) HaCaT, tumorigenic HPV- C-33A, tumorigenic HPV-18 CaLo, and tumorigenic HPV-16 SiHa cell lines irradiated with UVB. Cells’ irradiated proliferation was analyzed by crystal violet after exposure with increasing doses of UVB (220.5, 441, 661.5, 882 and 1102.5 J/m^2^) after 72 h of incubation in UVB-free conditions. Considerable change in proliferation was recorded at 661.5 J/m^2^ of UVB dose in all cell lines, although the intensity varied among them. Interestingly, C-33A and CaLo cells (Figure 1a,c) were more susceptible than HaCaT and SiHa cells (Figure 1b,d), suggesting tumorigenesis status and HPV type infection may determinate the cells’ UVB response.

Interestingly, the IC50 analysis showed important differential effects between immortal and tumorigenic, and HPV positive or negative cell lines (Table 1). It can be observed that C33A cells are more sensitive to UVB, with an IC50 of 378.3, followed by the CaLo cells with an IC50 of 474.8, suggesting HPV presence may play a role in cells’ susceptibility to UVB. HaCaT cells presented an IC50 of 681.9, suggesting lower sensitivity than tumorigenic C-33A and CaLo cells, and that tumorigenesis may favor UVB sensibility. Nevertheless, SiHa cells were less affected by UVB irradiation, with an IC50 of 724.7 J/m^2^. Therefore, it seems that cell context like HPV presence and type, as well as gene and protein expression, could influence UVB cell response. Cell proliferation inhibition elicited in these UVB-IR cells may be a consequence of cell cycle detention and/or cell death induction; therefore, we analyzed these processes to further characterize the UVB effect in cervical cancer cell lines.

### 2.2. UVB Inhibits Cell Cycle of Immortal, Tumorigenic and HPV Positive and Negative Cells in A Dose-Dependent Manner

**Figure 2:**
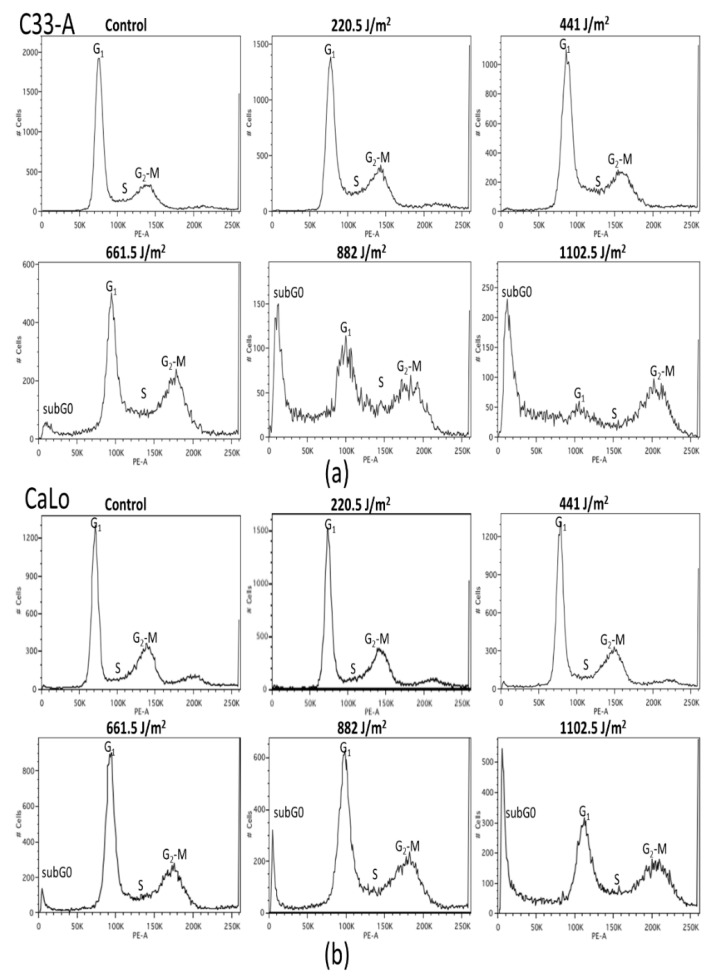

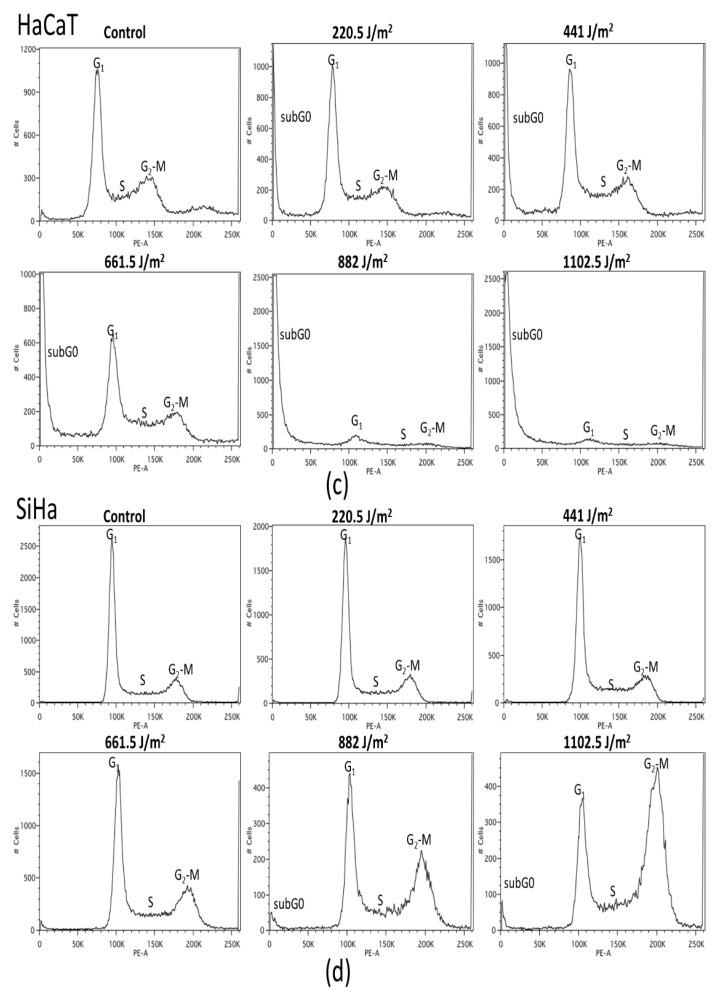
UVB inhibits cell cycle of cervical cancer cell lines in a dose-dependent manner. (**a**) C-33A, (**b**) CaLo, (**c**) HaCaT and (**d**) SiHa cells were treated with increasing doses of UVB and cell cycle was analyzed by flow cytometry after 72 h of incubation. Control cells were not UVB irradiated. Phases of cell cycle, G_1_, S and G_2_-M are shown in the figure. Sub-G0 phase is indicative of cell death.

Proliferation effect on different cell types would also be reflected on differential cell cycle progression elicited by UVB-IR. HaCaT, C-33A, CaLo and SiHa cells were treated with increasing doses of UVB and cell cycle was analyzed by flow cytometry after 72 h. Notably, a cell type-specific cell cycle effect was observed (Figure 2).

At 441 J/m^2^, arrest in S and G_2_ cell cycle phases was evident in HaCaT cells, while in C-33A and CaLo cells the effect was recorded at 661.5 J/m^2^ (Figure 2a–c) of UVB-IR. It should be noted that in HaCaT cells at higher UVB doses, characteristic cell cycle picks were not evident and cell death subG0 picks were evidenced since 220.5 J/m^2^, while in C-33A and CaLo cells, subG0 picks were noticed until 661.5 J/m^2^. Differently, SiHa cells registered S and G_2_ cycle arrest at 881 J/m^2^, showing resistance at lower UVB doses (Figure 2d). Interestingly, SiHa cell cycle histograms did not show many cells in subG0 phase. It is again noticeable that HPV presence or absence is not determinant to protect or sensitize cells to UVB damage, but HPV type could play a role in UVB resistance. All cell lines exhibited high amounts of cells held in the subG0 phase, suggesting cell death was induced along cell cycle arrest (Figure 2).

### 2.3. UVB Induces Apoptosis of Immortal, Tumorigenic and HPV Positive and Negative Cell Lines in A Dose-Dependent Manner

In the light of cell cycle data suggestion of cellular death, we explored apoptosis and necrosis processes in the cell lines exposed to UVB.

**Figure 3:**
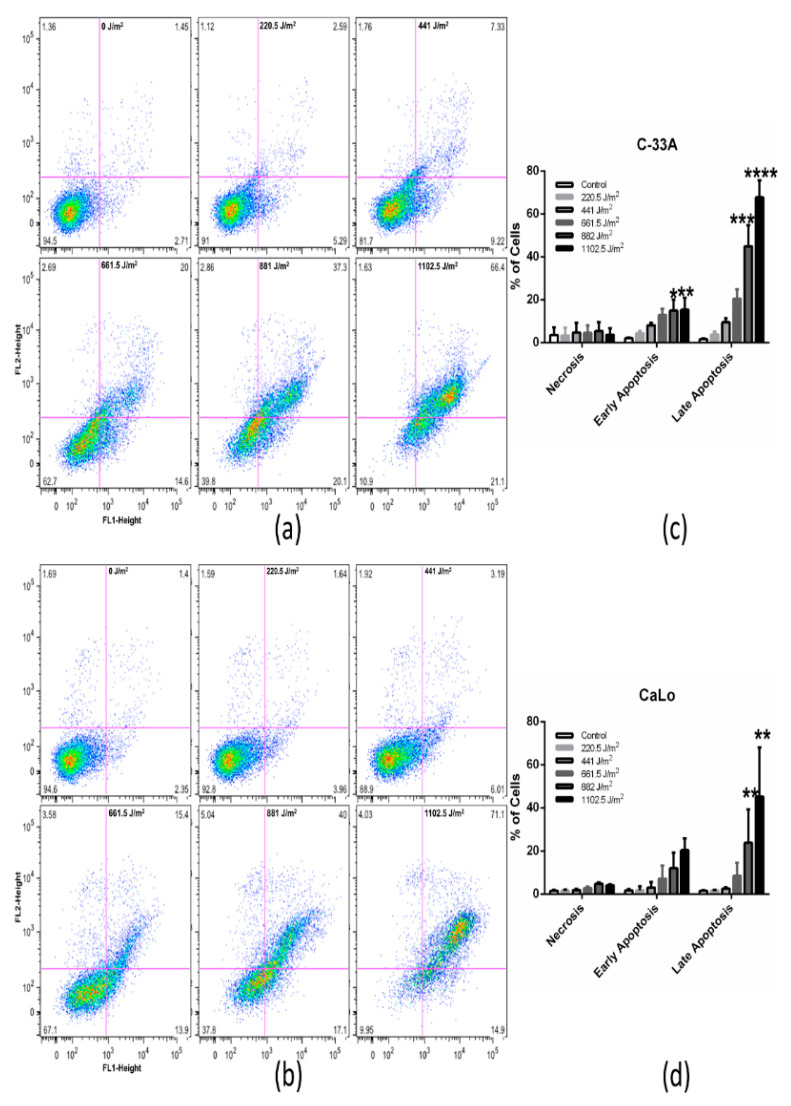

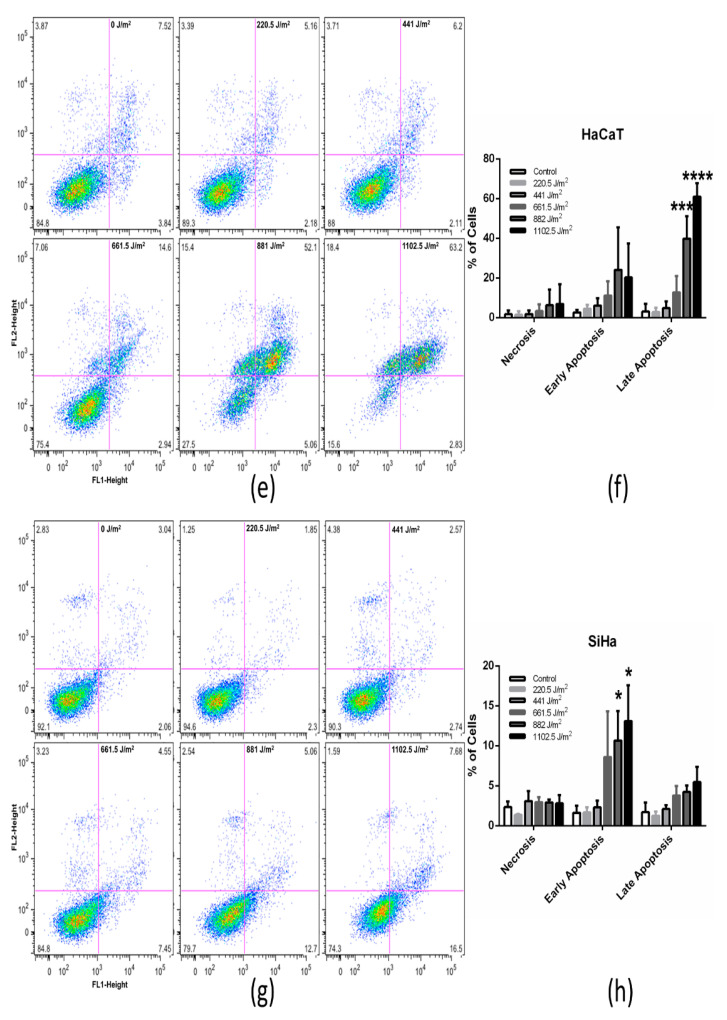
UVB differentially inhibits cell death of immortal and tumorigenic cell lines. Apoptosis and necrosis were analyzed by flow cytometry after 72 h of incubation in (**a**) C-33A, (**c**) CaLo, (**e**) HaCaT and (**g**) SiHa cells treated with increasing doses of UVB. The quantity of (**b**) C-33A, (**d**) CaLo, (**f**) HaCaT and (**h**) SiHa cells in early apoptosis, late apoptosis and necrosis are graphed. Cells in lower left quadrant are negative for Annexin-V and IP. Cells in upper left quadrant are necrotic cells. Cells in lower right quadrant are positive for Annexin-V and therefore are early apoptotic cells. Cells in upper right quadrant are positive for Annexin-V and IP and hence are late apoptotic cells. A one-way ANOVA multiple comparison with Tukey post-hoc test was performed. The bars represent the mean and standard deviation of three independent experiments (*p* < 0.05). Asterisk significance is as follows: * = 0.0143 at 882 and ** = 0.0048 at 1102.5 J/m^2^ in early apoptosis and *** = 0.0003 at 882 and **** = 0.0001 at 1102.5 J/m^2^ in late apoptosis in C-33A; ** = 0.0058 at 882 and 0.0046 at 1102.5 J/m^2^ in late apoptosis in CaLo; *** = 0.0003 at 882, and **** = 0.0001 at 1102.5 J/m^2^ in late apoptosis in HaCaT; *= 0.0261 at 882 and 0.0128 at 1102.5 J/m^2^ in early apoptosis in SiHa. Control cells were not UVB irradiated.

Noteworthy, UVB differentially induces cell death of immortal and tumorigenic cell lines. C-33A cells presented a null effect in necrosis while an increase in early (*p* < 0.0143 and 0.0048) and late apoptosis (*p* < 0.0003 and 0.0001) was recorded from low to higher doses of UVB (Figure 3a). On the other hand, HaCaT cells experienced a null effect of necrosis while late apoptosis was increased since 882 J/m^2^ (*p* < 0.0003 and 0.0001) (Figure 3c). Interestingly, in CaLo cells, an increase in necrosis and apoptosis was recorded at 882 and 1102.5 J/m^2^ (*p* < 0.0058 and 0.0046), while in SiHa cells, early apoptosis was observed since 882 J/m^2^ (*p* < 0.0261 and 0.0128), and cells either in late apoptosis or necrosis were not observed (Figure 3b,d). It is noteworthy to direct attention to SiHa cells’ resistance to UVB-IR in contrast to the other tumorigenic, immortal and HPV18 positive cell lines. This effect is probably due to the presence of HPV16 in this cell line, and/or other proteins involved in apoptosis regulation cell signaling.

### 2.4. p53, E2F1 and Microtubules System Proteins Are Regulated in Tumorigenic, HPV 16 Positive Cell Line SiHa Treated with UVB

**Figure 4:**
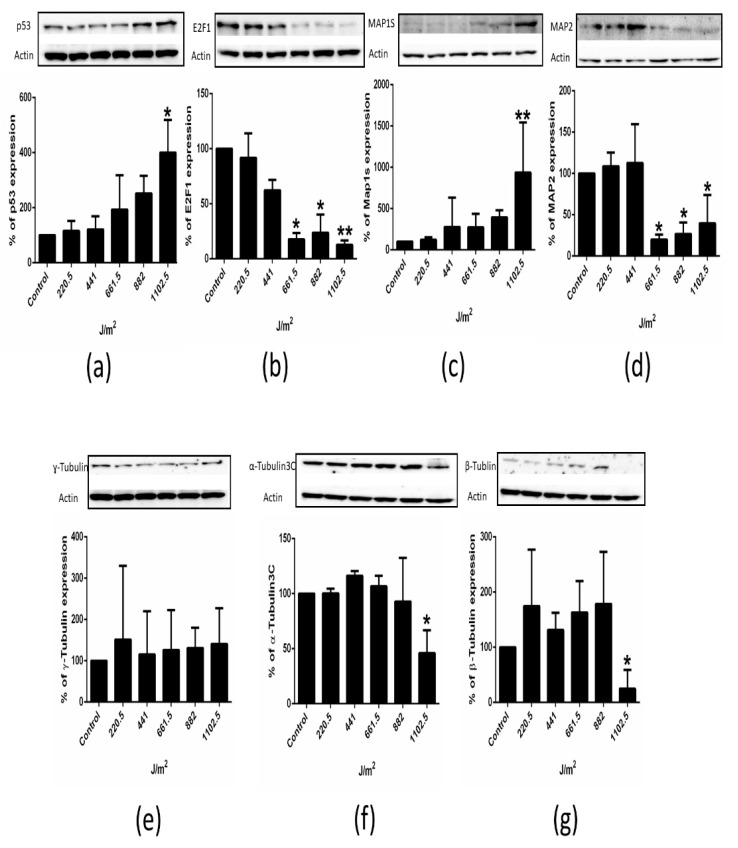
p53, E2F1 and microtubules system proteins are regulated in SiHa cells treated with UVB. SiHa cells were treated with increasing doses of UVB for 72 h and the expression of p53, E2F1 and proteins of microtubule system (MAP1S, MAP2, γ-Tubulin, β-Tubulin, α-Tubulin3C) were analyzed by Western blotting. (**a**) The protein expression of p53 was analyzed. Five independent experiments were analyzed by Kruskal–Wallis test in the bottom graphic (1102.5 J/m^2^ * *p* < 0.0332). (**b**) The protein expression of E2F1 was analyzed. Five independent experiments were analyzed by Kruskal–Wallis test (661.5 J/m^2^ * *p* < 0.0374), 882 J/m^2^ *(*p* < 0.0473) and 1102.5 J/m^2^ ** (*p* < 0.0031). (**c**) The protein expression of MAP1S was analyzed. Five independent experiments were analyzed by Kruskal–Wallis test (1102.5 J/m^2^ * *p* < 0.0094). (**d**) The expression of MAP2 was analyzed. Three independent experiments were analyzed by correlation test (*r* = −0.6754 and *p* < 0.0021). (**e**) The protein expression of γ-Tubulin was analyzed. Three independent experiments were analyzed by correlation test (*r* = −0.7674, *p* < 0.0002). (**f**) The protein expression of α-Tubulin3C was analyzed. Three independent experiments were analyzed by correlation test (*r* = −0.4932, *p* < 0.0375). (**g**) The protein expression of β-Tubulin was analyzed. Three independent experiments were analyzed by correlation test (*r* = −0.506, *p* < 0.0322). The protein expression of p53, E2F1, MAP1S, MAP2, γ-Tubulin, β-Tubulin and α-Tubulin3C were analyzed comparing control cells to exposed irradiated cells. Control cells were not UVB irradiated.

We showed that UVB induced proliferation inhibition, cell cycle arrest and apoptosis in a dose-dependent manner. These cell processes are conventionally regulated by several key proteins such as p53 and E2F1; therefore, we evaluated their regulation in UVB-IR SiHa cells. Additionally, we analyzed the expression of various proteins related to microtubules system that have not been examined in this cell line by Western-blotting technique. According to apoptosis induction, an increase in p53 protein was evident since 881 J/m^2^ (Figure 4a). Interestingly, E2F1 protein expression decreased since 661.5 J/m^2^ and continued its down regulation to 1102.5 J/m^2^ (Figure 4b), suggesting p53-independent and E2F1-dependent regulation was taking cell cycle control since lower UVB dose. On the other hand, MAP1S protein expression considerably increased at the higher dose of 1102.5 J/m^2^ (Figure 4c), while the protein expression of MAP2 showed a dual expression—at 441 J/m^2^ it was shown to have increased while it considerably decreased at 661.5 to 1102.5 J/m^2^ (Figure 4d). γ-Tubulin overexpression has been previously observed in different cancer types; surprisingly, γ-Tubulin expression under these conditions was found increased since 882 J/m^2^ and remained at similar levels at 1102.5 J/m^2^ (Figure 4e). However, this increment was not statistically significant, probably indicating γ-Tubulin regulation is not determinant to the cellular processes regulated under our conditions of UVB exposure of SiHa cells. Regarding other tubulins, the expression of α-Tubulin3C was observed as decreased only until 1102.5 J/m^2^ (Figure 4f), while the protein expression of β-Tubulin showed a dual pattern: at 661.5 J/m^2^, its expression considerably started to increase while it was markedly reduced at 1102.5 J/m^2^ (Figure 4g), suggesting β-Tubulin differently participates in cell processes’ regulation after different levels of cell damage. In light of the present results, it is appropriate to speculate that E2F1 and MAP2 proteins are regulated under lower doses of UVB and the rest of the proteins analyzed are regulated under more severe stress conditions and are probably the consequence of various systems activated upon radiation exposition.

## 3. Discussion

UVB has important activity in human physiology [19,20]. High irradiation and time exposure of UV can conduce to cancer development as well as its prevention [21]. Given this dual effect of UV, in the present work we explored UVB effect on different HPV negative and positive cervical tumorigenic cell lines as well as immortal HPV negative keratinocyte cells. UVB lethality in cells of several kinds has been shown to be dose- and time exposure-dependent [6,7]. The participation of p53 in UVB-mediated cell death has been previously reported [22]. In our present work, it was also shown that p53 protein plays a role in UVB-induced apoptosis in SiHa cells. Interestingly, mutated or down-regulated p53 is usually found in SiHa cell line [23], suggesting wild type p53 is also present in this cell line or it is strongly induced after DNA damage. Cell proliferation, cell cycle and apoptosis processes were affected by UVB in all cells assayed in our work. Furthermore, we observed a differential UVB effect, HPV content and tumorigenic phenotype response in a dose-dependent manner. Noteworthy, a clear resistance was observed in SiHa cells; however, additional experiments are needed to elucidate if this resistance is the consequence of HPV16 presence or if it is due to cellular gene and protein expression and/or cell signaling response to UVB. Further protein expression analysis of UVB resistant SiHa cells showed that E2F1 and MAP2 proteins are early down-regulated upon UVB-IR at 441 J/m^2^ and 661.5 J/m^2^, respectively, probably eliciting proliferation inhibition and cell cycle arrest. Other microtubule system proteins were modified at their expression only under more severe cellular damage, including MAP1S, α-Tubulin and β-Tubulin, whose expression was increased at 661.5 and 1102.5 J/m^2^, respectively. This protein regulation was also observed for p53 protein expression correlated with cell cycle arrest and apoptosis increase, probably indicating an active participation in these processes at later stages. It has been reported that UVB treatment inhibits proliferation, induces cell cycle arrest and apoptosis partially p53 responsible via p21, Growth Arrest and DNA Damage-inducible (GADD45) and BCL2-associated X protein (BAX) increased expression [22]; however, a p53-independent effect must not be discarded [7]. E2F1 response to UVB analysis has shown increased E2F1 expression and quiescent cells entering cell cycle S phase and proliferation induction [24,25]. Noteworthy, increased E2F1 expression has been observed under short UV exposure [26]. Nevertheless, in our work, a reduced expression of E2F1 was observed, probably attributable to wild type p53 expression in SiHa cells, as has been previously shown [27]. P53 and E2F1 interaction currently could explain some of the effects in UVB-treated cells. However, it should be noted that other RNAs and/or proteins could be relevant in the observed UVB response and could be complementary to each other. In this sense, it has been reported that centrosome and γ-Tubulin interact with E2 promoter binding factors to regulate E2F transcriptional activity controlling cell cycle progression. γ-Tubulin interacts with transcriptionally active E2F1 during G1/S transition. Therefore, transcriptional activity of E2F is altered by reduced expression of γ-Tubulin. γ-Tubulin C terminus encodes a DNA-binding domain that interacts with E2F-regulated promoters, resulting in γ-Tubulin-mediated transient activation of E2Fs [28]. In our present work, we observed a slight γ-Tubulin increase. Nevertheless, it should be noted that E2F1 was down-regulated after UVB exposure, probably via pRB regulation, as was shown by Ehlen et al. 2012 [11]. Interestingly, in our study, γ-Tubulin expression is accompanied with a slight increase at low doses and decreased expression of β-Tubulin and α-Tubulin3C in SiHa cells at higher doses of UVB. It seems that β-Tubulin and α-Tubulin3C expression are altered in response to several cell signaling pathways associated to UVB programming gene expression. It has been reported that MAP1S stabilizes microtubules and is involved in spindle formation [29]. We observed that MAP1S responded at 882 and 1102.5 J/m^2^ doses of UVB treatment in SiHa cells, suggesting its possible implication in apoptosis via mitochondria regulation [17] and/or proliferation inhibition via mitotic arrest [29]. Differently, MAP2 protein expression was down-regulated by UVB-IR in SiHa cells. MAP2 has been observed with cell migration and invasion in epithelial and oral cancer cells [16]. MAP2 can serve as an architectural element by establishing specific morphological features and specific arrangements of microtubules [30]. Defects in segregating chromosomes and positioning the nucleus during mitosis can lead to proliferation inhibition and cell death in tumor cells [18]. Therefore, the observed proliferation inhibition, S-arrest and apoptosis may all be a consequence of microtubule dysfunction produced by microtubule-binding proteins. The present data provide evidence of protein response to UVB-IR in cell proliferation, cell cycle and apoptosis in SiHa cells. In addition, E2F1 and MAP2 are suggested as molecular targets for UVB first line of response that could be used to monitor proteins altered in genotoxic stress and/or molecular targets in cancer. Further experiments are needed to elucidate the mechanism underlying the signaling that triggers a differential protein expression in cervical carcinoma cell lines. Our results suggest that E2F1 and MAP2 are proteins that early respond to UVB while MAP1S, p53 and tubulins are proteins that play a role later in UVB-induced cell damage and cell signaling.

## 4. Materials and Methods

### 4.1. Cell Lines

SiHa (cells were originally obtained from a cervical cancer tumor of a 55 years old Asian female adult), CaLo (cells were obtained from a cervical cancer tumor of a 55 years old Mexican female adult), C-33A (cells were originally obtained from a cervical cancer tumor of a 66 years old Caucasian female adult) and HaCaT (were originally obtained from keratinocytes of a 62 years old male adult) cells were grown in Dulbecco’s Modified Eagle’s Medium (Invitrogen Corporation, Carlsbad, CA, USA) enriched with 5% fetal bovine serum. Medium change and passage were performed every 3 and 4 days, respectively. All cell lines were kindly provided by Gariglio’s Lab from CINVESTAV-IPN.

### 4.2. UVB Treatment of Cell Lines

Cells were counted in a Neubauer chamber using an inverted microscope to seed 2 × 10^5^ per well and irradiated since 220.5 until 1102.5 J/m^2^ using a spectroline ultraviolet transilluminator followed by 72 h of incubation at 37 °C and 5% CO_2_ at 319 nm.

### 4.3. Cell Proliferation Analysis

Cell proliferation was quantified by crystal violet dye in 1XPBS. The treated cells were incubated in methanol for 15 min and washed two times with distilled water. Cells were dyed with 0.1% crystal violet and washed three times with water, and finally crystal violet was recovered with 10% acetic acid to be analyzed in microplate reader Multiskan GO Spectrophotometer (Thermo Scientific™, Ratastie, Finland).

### 4.4. Cell Cycle Analysis

Treated cells were harvested at 72 h and fixed in 80% ethanol at −20 °C overnight before incubation with 30 µg/mL DNase-free RNaseA (Thermo Scientific™) and stained with 6 µg/mL propidium iodide (Sigma–Aldrich, St. Louis, MO, USA) for 1 h. Samples were acquired in a FACS Canto II flow cytometer (Becton Dickinson, CA, USA). Data analysis was carried out using Flow Jo (FlowJo 7.6.2 Software; FlowJo LLC, Ashland, OR, USA).

### 4.5. Apoptosis Analysis

Cells for apoptosis analysis were stained using Annexin-V-FLUOS Kit as described by the manufacturer (Roche Diagnostics GmbH, Penzberg, Germany). Samples were acquired in a FACS Canto II flow cytometer (Becton Dickinson). Data analysis was carried out using Flow Jo (FlowJo 7.6.2 Software; FlowJo LLC, Ashland, OR, USA).

### 4.6. Immunoblotting

Cells were washed with 1X PBS and lysed in RIPA buffer, and 50 μg of total soluble proteins were denatured in Laemmli loading buffer and boiled for 5 min before gel electrophoresis using 10% polyacrylamide/SDS. Gels were electro-transferred to supported nitrocellulose membrane (Bio-RAD, Hercules, CA, USA) and incubated with primary antibodies for α-Tubulin3C (sc-134240), β-Tubulin (sc-5274), γ-Tubulin (17787), MAP1S (324102), E2F1 (sc-251), p53 (Sc-126), MAP-2 (sc-20172) and β-Actin (sc-1616) (Santa Cruz Biotechnologies, Santa Cruz, CA, USA). Primary and secondary antibodies were used at 1:1000 and 1:15000 dilution, respectively. Detection was performed using Western Blotting Luminol Reagent sc-2048 (Santa Cruz Biotechnology, TX, USA) and analyzed by Imagelab Software of Gel Doc TM XR ChemiDoc TM XRS, Universal Hood II, (Bio-RAD, Hercules, CA, USA).

### 4.7. Statistical Analysis

Data normality was verified through D′Angostino–Pearson tests. Depending on normal distribution of the data, multiple comparisons one-way ANOVA or Kruskal–Wallis tests were performed. For the identification of the specific differences, Tukey or Dunn post tests were performed accordingly. Correlation analysis was also performed using Pearson or Spearman correlation analysis according to data distribution. A level of alpha was established at <0.05. All analysis was carried out using GraphPad Prism Software V.6 [31].

## 5. Conclusions

Immortal HPV-HaCaT cells were more resistant to UVB irradiation than tumorigenic HPV-C-33A and HPV18 CaLo cells in cellular processes associated with cancer progression including proliferation, cell cycle and apoptosis, suggesting tumorigenic status may favor UVB-induced cell death. Noteworthy, the tumorigenic HPV16 SiHa cells were more resistant to UVB radiation than HaCaT cells, probably due to HPV16 content that could modify cell signaling and gene expression. The use of UVB as a treatment could be selectable for HPV18 infections or HPV18 positive cancer; however, more studies are needed to elucidate its possible application in HPV illness-related treatment. Surprisingly, in SiHa cells, the expression regulation of p53, a well-UV-responsive element, was evidenced later after UVB treatment than the expression change of E2F1, a cell cycle regulating protein, probably due to HPV16 E6 protein p53 regulation being stronger than HPV16 E7 protein effect on regulating E2F1 activation, or this may be due to other induced pathways involving p53-independent ways of cell cycle regulation and/or apoptosis. Proteins of the microtubules system also presented different dose-dependent responses. On one hand, MAP1S expression was increased since 882 J/m^2^, while MAP2 presented a dual pattern expression—at low doses its expression was apparently increased whereas at higher doses it was considerably decreased. α- and β-Tubulin expression decreased at final UVB doses while γ-Tubulin remained constant. Taken together, our results suggest that E2F1 and MAP2 genes are first UVB responders, p53 and MAP1S are strongly regulated after higher irradiation doses and α-Tubulin and β-Tubulin are the last proteins being regulated after stronger UVB-induced cellular damage. This study adds to the knowledge of the effect of a specific band of UV (UVB) on cancer cellular processes of cervical cancer cell lines with different HPV content, highlighting some proteins involved in such effects to validate the possible application of UVB in the treatment of cervical cancers with specific HPV content.

## Figures and Tables

**Table 1:** IC50 of cells UVB-IR and cell characteristics.

Cell Line	HPV Status	Cell Phenotype	IC50
C-33A	HPV negative	Tumorigenic	378.3 J/m^2^
CaLo	HPV 18 positive	Tumorigenic	474.8 J/m^2^
HaCaT	HPV negative	Immortal	681.9 J/m^2^
SiHa	HPV 18 positive	Tumorigenic	724.7 J/m^2^

## Data Availability

Data is contained within the article.
